# PNPLA3-I148M: a problem of plenty in non-alcoholic fatty liver disease

**DOI:** 10.1080/21623945.2019.1607423

**Published:** 2019-05-07

**Authors:** Soumik BasuRay

**Affiliations:** Eugene McDermott Center for Human Growth and Development, Department of Molecular Genetics, University of Texas Southwestern Medical Center, Dallas, TX, USA

**Keywords:** Fatty liver disease, lipid droplet, PNPLA3, proteasome, autophagy

## Abstract

Fatty liver disease (FLD) affects more than one-third of the population in the western world and an increasing number of children in the United States. It is a leading cause of obesity and liver transplantation. Mechanistic insights into the causes of FLD are urgently needed since no therapeutic intervention has proven to be effective. A sequence variation in patatin like phospholipase domain-containing protein 3 (PNPLA3), rs 738409, is strongly associated with the progression of fatty liver disease. The resulting mutant causes a substitution of isoleucine to methionine at position 148. The underlying mechanism of this disease remains unsolved although several studies have illuminated key insights into its pathogenesis. This review highlights the progress in our understanding of PNPLA3 function in lipid droplet dynamics and explores possible therapeutic interventions to ameliorate this human health hazard.

## Introduction

Fatty liver disease is a burgeoning health hazard that affects more than one-third of the population in the western world [1]. It is divided into two types: non-alcoholic and alcoholic. Non-alcoholic fatty liver disease (NAFLD) affects ~25% population worldwide and is most prevalent in the Middle East and South America with the lowest incidence in Africa [2].

NAFLD is typically associated with obesity and insulin resistance [3,4]. Its progression is characterized by four stages: Steatosis (first) leading to non-alcoholic steatohepatitis (NASH), a condition characterized by inflammation and ballooning (second). This condition may develop into organ impairment or cirrhosis (third) leading to the end stage of hepatocellular carcinoma (fourth) necessitating liver transplantation [5]. NAFLD will soon overtake Hepatitis C as the leading indication of liver transplantation [6].

NAFLD has multifactorial pathogenesis involving a close interplay of environmental factors and genetic determinants. There are numerous studies, suggesting ethnic difference as the major cause of hepatic fat [7,8]. The first clinical evidence of association of a variant of the *PNPLA3* (aliases Adiponutrin, calcium-independent phospholipase A2 epsilon) gene, rs738409 C > G with NAFLD development was provided by Romeo *et.al* who demonstrated that the frequency of the PNPLA3-I148M variant was significantly higher in the Hispanics (49%) compared to the European Americans (23%) and African Americans (17%). The prevalence of hepatic steatosis as measured by proton magnetic spectroscopy was higher in the Hispanics (45%) than European Americans (33%) and African Americans (24%) [4]. This variant was also associated with alcoholic liver disease [9] and accumulation of hepatic fat in studies across different ethnicities and geographical region [10].

*Pnpla3* was first cloned from a cDNA library of 3T3 preadipocytes during differentiation into mature adipocytes and hence was named ‘adiponutrin’ [11]. In mice, *Pnpla3* is highly expressed in white and brown adipocytes and modestly expressed in the liver (Ct = 25).In mice, this gene is nutritionally regulated in response to carbohydrate and insulin treatment [11–13]. In humans, however, its expression is 10-fold higher in the liver than adipose tissue [14]. Nutritional regulation of PNPLA3 is robust in humans similar to mice. Low-calorie diet reduces PNPLA3 expression in the adipose tissue that gets upregulated on refeeding [15] by both insulin and glucose [16]. In liver, PNPLA3 is positively associated with body mass index(BMI) [17]. Thus,PNPLA3 demonstrates nutritional regulation.

## Tissue distribution and nutritional regulation

PNPLA3 is one among the nine members of patatin like phospholipase domain-containing proteins (PNPLA1-9) [18]. Patatin is a major protein of potato tuber with non-specific lipid acyl hydrolase activity [19,20]. It is a storage protein but cleaves fatty acids from membrane lipids by its lipase activity [21].

PNPLA3 is variedly expressed in human and mice. In humans, *PNPLA3* is highly expressed in the liver and retina. In the liver, it is expressed in hepatocytes, stellate cells and sinusoidal cells [22–26]. In mice, *Pnpla3* is expressed in brown and white adipose tissues, adrenal gland, skeletal muscle, heart and liver. Unlike humans, its expression is higher in the adipose tissue compared to liver [11,27]. In the liver, *Pnpla3* levels are higher in hepatocytes than stellate cells [23]. The PNPLA3-I148M do not show an association of high liver fat content with insulin resistance [4,28]. Elevated diacyl glycerol (DAG) is associated with insulin resistance in rodents and humans with steatosis yet PNPLA3-I148M is not associated with changes in insulin sensitivity [29]. This disconnect is due to the unaltered proportion of DAG (FA 18:1) in PNPLA3-I148M carriers [30].Irrespective of differential tissue expression, murine models have provided significant insights into the pathogenesis of NAFLD caused by PNPLA3-I148M mutant. In mice, overexpression of the human PNPLA3 and PNPLA3-I148M in adipose tissue did not reveal any change in morphology or function of either white or brown fat or cold tolerance. The increased liver fat was associated with only liver specific overexpression of PNPLA3-I148M suggesting that the fatty liver phenotype is associated with the disease mutant in liver rather than the adipose tissue [31]. However, this model has limitations. The sequence homology of human and mice PNPLA3 is 68%. The human PNPLA3 is 481 amino acid in length while the mouse PNPLA3 is 384. The extended human PNPLA3 has two vesicle targeting motifs [32]. The transgenic mouse constitutively overexpresses human PNPLA3 and is not nutritionally regulated. To overcome these shortcomings a knock-in (KI) model was developed by Smagris *et al*., that developed steatosis on high sucrose challenge with pronounced accumulation of the mutant at hepatic lipid droplets [33].*Pnpla3* is nutritionally regulated. It is reduced on fasting and robustly expressed on high carbohydrate refeeding by insulin [11,23,27,34]. Previous studies have shown that PNPLA3 is located at the lipid droplets and the PNPLA3-I148M is associated with droplets of larger size with reduced triglyceride(TG) hydrolysis [35–37]. Biochemical fractionation studies have indicated that more than 90% of the cellular PNPLA3 pool resides at the lipid droplets [35]. Thus, PNPLA3 is a predominantly lipid droplet resident protein.

In mice, the wild type PNPLA3 is undetectable 6h post fasting whereas this effect this blunted and the protein persists till 12h fasting in PNPLA3-I148M KI mice due to reduced ubiquitylation and impaired proteasome targeting [38]. It is possible that the disease variant alters the conformation of PNPLA3 and reduces the access of E3 ligase. The mutant might trap the substrate in a conformation unrecognizable by the E3 ligase or it may be rapidly deubiquitylated thereby reducing turnover. This makes it imperative to identify the ubiquitylation sites of PNPLA3 to explore therapeutic opportunities.

In mice, hepatic *Pnpla3* is regulated under the transcriptional control of SREBP1c (Sterol regulatory binding protein 1c) and ChREBP (Carbohydrate response element binding protein) [39]. In humans, two independent studies have shown that it is regulated exclusively by SREBP1c [23,39] while another group reported its regulation by glucose through ChREBP [40]. SREBP1c is ubiquitylated and targeted to the proteasome by E3 ligase Ring Finger Protein 20 (RNF20) to regulate hepatic lipid metabolism [41]. Transcriptional program helps to maintain long-term controls, but deactivation or degradation of excess protein is an added layer of lipid homeostasis.PNPLA3 is under acute nutritional control possibly by post-translational modification that fine-tunes its regulation.

## PNPLA3 function

PNPLA3 has been shown to have different enzymatic activities. PNPLA3 expressed in Sf9 insect cells demonstrated TG lipase and acylglycerol transacylase activities [42]. Similar activities were reported in a cell-free system [27]. In 2011, Huang *et al*. reported that PNPLA3-I148M mutant showed an impaired TG lipase activity suggesting a loss of function in the development of steatosis. In the same report, they did not observe acyltransferase activity of either the PNPLA3 wild-type or PNPLA3-I148M mutant [43]. However, in an independent study trigger factor fused soluble PNPLA3 was shown to have a lysophosphatidic acid acyl transferase (LPAAT) activity which increased when PNPLA3-I148M was overexpressed, suggestive of a gain of function mutation [44]. However, a subsequent study failed to detect any increase in TG biosynthesis in the PNPLA3-I148M KI mice model of hepatic steatosis arguing against such a possibility [38]. A possible explanation for this discrepancy is since the proteins were purified from *E.coli* it likely co-purified an endogenous LPAAT as reported in a subsequent study [45]. Purified PNPLA3 and PNPLA3-I148M from *Pichia pastoris* (yeast) showed robust TG hydrolase and modest acyltransferase activities [46].

PNPLA3 is well expressed in human liver and retina necessitating further studies on its activity towards retinyl esters in human stellate cells. Purified PNPLA3 showed retinyl palmitate hydrolase activity which was impaired in the PNPLA3-I148M mutant leading to massive accumulation of retinyl esters in these cells [24]. Humans harbouring the PNPLA3-I148M mutation were found to have lower levels of circulating retinol and retinol binding protein 4 and had a higher content of hepatic retinyl esters suggesting a role of PNPLA3 in regulating the release of retinol from stellate cells [25,47].

PNPLA3 has also been suggested to play a role in lipid remodelling in hepatic TGs. Human hepatocytes expressing PNPLA3 and PNPLA3-I148M had higher levels of very long-chain polyunsaturated fatty acids (PUFA) in the phospholipids. The PNPLA3-I148M mutant was associated with lower levels of arachidonic acid in hepatic TGs [37,48,49]. Consistent with these reports, Mitsche *et al.*, reported that PNPLA3 and PNPLA3-I148M transfer PUFA from TGs to phospholipids. Arachidonic acid was the major lipid transferred to phospholipids in hepatic lipid droplets of PNPLA3-I148M KI mice. This function was not observed in the catalytically dead PNPLA3 –S47A mutant and PNPLA3 knockout mice [50]. Arachidonic acid is also the major substrate of membrane-bound – o acyl transferase domain containing protein7 (MBOAT7) [51], a mutant of which is implicated in alcoholic cirrhosis [52]. In sum, phospholipid remodelling and NAFLD susceptibility seem to go hand in hand.

Studies in mice models have shed considerable light on PNPLA3 function. These reports indicate that the PNPLA3-I148M is neither a gain nor loss of function mutation but a neomorph. Animal models of *Pnpla3* knockout do not exhibit hepatic TG accumulation hence do not develop steatosis [53,54]. Experiments in transgenic mice models argue against a gain of function of PNPLA3-I148M mutant. Mice overexpressing human wild type PNPLA3 have TG levels similar to the non-transgenic mice, although PNPLA3-I148M overexpression recapitulates the human steatosis phenotype [31].

PNPLA3 shares most sequence homology (~46%) with PNPLA2 or ATGL that plays a crucial role in the rate-limiting step of TG hydrolysis [55]. Unlike other lipases that have catalytic triad (Ser-His-Asp), patatins use a catalytic dyad (Ser-Asp). Ser47 is a conserved residue of the hydrolase motif (Gly-X-SerX-Gly) that lies between a beta strand and an alpha helix. The crystal structure of the heartleaf horse nettle patatin is similar to the homology modelling of PNPLA3 [19,35]. Two contrasting views are in the field regarding the PNPLA3 structure. There are reports suggesting that PNPLA3 has membrane-spanning domains on the basis of secondary structure prediction [11] with a strong association with endoplasmic reticulum and lipid droplets [27,42] yet other reports of homology modelling of PNPLA3 suggest the alpha helixes to be part of the globular structure precluding the membrane span [35]. The crystal structure of PNPLA3 is yet to be determined.

## Degradation pathways of PNPLA3

Murine models of fatty liver have unravelled significant insights into the mechanistic basis of PNPLA3-I148M induced steatosis. PNPLA3 is ubiquitylated and targeted to the proteasome [23]. The mutant PNPLA3-I148M as well as the catalytically dead PNPLA3-S47A accumulate at the hepatic lipid droplets on high sucrose feeding in KI mice [33]. PNPLA3-I148M continues to sustain at lipid droplets on prolonged fasting due to impaired ubiquitylation and proteasome targeting [38]. It is possible that the mutant undergoes a conformational change that restricts access of the E3 ligase. Both PNPLA3-I148M and PNPLA3 –S47A impair the catalytic activity of the enzyme by reducing its V_max_ and neither impairs substrate binding [43]. It is possible that the uncleaved substrate traps the enzyme in a conformation inaccessible to the E3 ligase. An alternative possibility is that both these mutants are rapidly deubiquitylated and stabilized on the droplets. Binding to lipid droplets inhibit proteasomal degradation of several lipid droplet proteins [56,57]. *In vivo* inhibition of proteasome by Bortezomib (8 h) showed that the levels of PNPLA3 could not match PNPLA3-I148M in the transgenic mice suggesting the contribution of other degradation pathways in its turnover. Although inhibition of macroautophagy by 3-methyladenine failed to elicit an increase in the wild type protein, it may be due to partial blockade of this pathway as reported earlier [58]. Involvement of chaperone-mediated autophagy as observed for other lipid droplet proteins cannot be ruled out [59].

Human genetic studies indicate that accumulation of PNPLA3-I148M mutant is required for the development of NAFLD [25]. A naturally occurring PNPLA3 polymorphism, rs2294918, E434K variant was linked to reduced hepatic PNPLA3 protein abundance [25]. Carriers of the PNPLA3 I148M- K434 variant did not predispose to liver damage in contrast to the risk variant I148M-K434E [25]. Although it is not known if K434 gets ubiquitylated, predictive algorithms suggest this residue to be a good candidate [60]. Human PNPLA3 has 19 lysine residues while the truncated mouse protein has 12. The K434 residue is absent in mouse PNPLA3. It is possible that PNPLA3 gets ubiquitylated at multiple lysine residues at the patatin domain and downstream at the C-terminus. A recent report showed K100 residue of PNPLA2/ATGL to be the main site of ubiquitylation with COPI as its ubiquitin ligase [61]. This residue is conserved in human PNPLA3. Three other residues in the patatin domain: K92, K135 and K179 are conserved in human and mouse PNPLA3 as well as human and mouse ATGL. All the above lysine residues are potential ubiquitylation sites.

A recent report suggests a lysine independent cysteine ubiquitylation of ACAT2 regulated by cholesterol and fatty acids [62]. PNPLA3 can have a similar mechanism of ubiquitylation. Human PNPLA3 has 18 cysteine residues while mouse PNPLA3 has 17 that could serve as potential ubiquitylation sites. In sum, identification of specific ubiquitylation sites is needed to determine the mechanism of evasion of ubiquitylation by the disease mutant.

In addition to the proteasome, PNPLA3 is also targeted to the autophagy pathway [63]. This has been demonstrated *in vitro* and *in vivo* by pharmacological intervention and knockdown of a key component (ATG7) of macroautophagy [64]. This is congruent with findings on the interaction of ATGL with LC3 through its conserved LC3 interacting region (LIR) motif to modulate lipophagy [65]. PNPLA3 has also been suggested to play a role in lipophagy [63] although no change in hepatic TG levels were observed in PNPLA3 knockout mice [64]. The role of PNPLA3 in autophagy and specifically hepatic lipid metabolism remains obscure and warrant further studies.

## Therapeutic strategies

NAFLD is a complex disorder and therapeutic options are limited. To date, no drugs have been approved by the Food and Drug Administration (FDA) for its treatment [66]. PNPLA3-I148M causes fatty liver. If the mechanistic basis for steatosis is similar in mice and humans then therapeutic interventions that lower PNPLA3-I148M protein levels are likely to be beneficial in ameliorating the condition in carriers with this variant.

**Figure 1.**
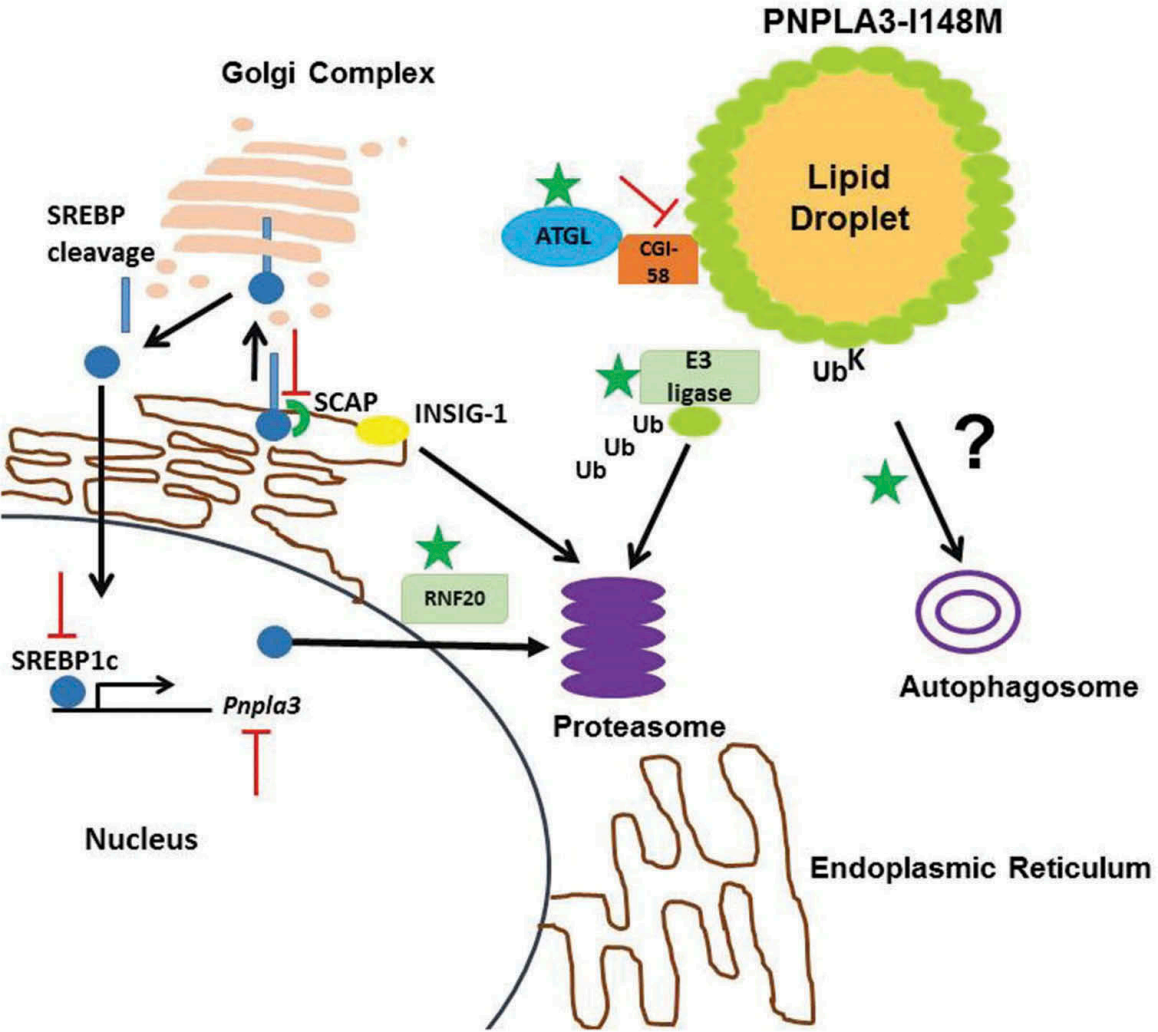
Potential drug targets for PNPLA3-I148M induced NAFLD. PNPLA3 is robustly expressed on feeding by upregulation of SREBP1c which gets escorted by SCAP from the endoplasmic reticulum to Golgi complex to be cleaved by S1 proteases. Insig-1 that prevents binding of SCAP to SREBPs gets degraded at the proteasome in the process. The mature form of SREBP1c translocates to the nucleus to upregulate *Pnpla3* expression. SREBP1c is degraded at the proteasome by its E3 ligase RNF20. PNPLA3 is a known substrate for proteasomal degradation although its specific E3 ligase is unknown. It is also known to be targeted to autophagy. Activation of proteins marked by 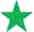 and inhibition marked by 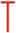 are attractive drug targets for PNPLA3-I148M induced fatty liver.

PNPLA3-I148M accumulation is a prerequisite for steatosis [64]. PNPLA3 function and its altered activity in the disease variant have thrown up many attractive drug targets (Figure 1). Downregulation of *Pnpla3* with antisense oligo nucleotides in rats resulted in 20% hepatic fat reduction and enhanced insulin sensitivity [67]. Genetic suppression of *Pnpla3* is an attractive strategy for regulating the protein expression in PNPLA3-I148M carriers. This strategy has been demonstrated to be effective in ameliorating fibrosis and steatosis in PNPLA3-I148M KI mice [64,68]. PROTAC3 mediated degradation of Halo tagged PNPLA3-I148M significantly reduces hepatic TG in mice challenged with high sucrose diet [64]. This therapeutic approach could be potentially useful in NAFLD patients harboring the PNPLA3-I148M variant.

Under fasting, insulin levels are low. As a result, the *SREBP-1c* gene is not actively transcribed, nuclear SREBP-1c levels are low and Insig-1 mRNA and protein levels are low. In contrast, Insig-2 levels are high, owing to the Insig-2a transcript [69]. *Pnpla3* is robustly expressed by SREBP1c on insulin mediation [70]. Insulin also induces *SREBP-1c* transcription, nuclear SREBP-1c activates the *Insig-1* gene, and Insig-1 mRNA and protein levels rise to higher levels than in the basal nonfasting state. Insig-2 gets replaced by Insig-1 upon refeeding [69]. Developing SREBP1c inhibitor can specifically modulate PNPLA3 expression at the transcriptional level. SREBP1c cleavage is activated by SREBP cleavage-activating protein (SCAP) [71]. Inhibition of SCAP activity could provide a broad spectrum effect in ameliorating steatosis [64]. Determination of E3 ligase(s) of PNPLA3 will help design small molecule activators to specifically target the accumulated protein for proteasomal degradation [64].

It remains to be tested if competition for CGI-58 between ATGL and PNPLA3-I148M reduces the fraction of ATGL bound CGI-58 in PNPLA3-I148M KI mice. Data from transgenic mice argue against this hypothesis [72,73]. Interaction of CGI-58 and ATGL is well documented, and knockout of liver CGI-58 results in steatohepatitis and fibrosis in mice [74,75]. PNPLA3 was shown to be colocalized with CGI-58 [36]. A recent report suggests that the pro-steatotic effect of PNPLA3 requires the presence of CGI-58 consistent with a model where PNPLA3-I148M promotes sequestration of CGI-58 from ATGL to impair its lipase activity at the lipid droplets [76]. It is possible that during fasting, CGI-58 is sequestered to the PNPLA3-I148M enriched lipid droplets to reduce the available pool for ATGL. The ubiquitin defective PNPLA3-I148M mutant accumulates at hepatic lipid droplets even after 12 h of fasting concomitant with CGI-58 [38]. PNPLA3-I148M interacts with CGI-58 possibly to impair ATGL activity as the basis of steatosis. If PNPLA3-I148M sequesters CGI-58 away from ATGL then perturbing this interaction with small molecules can prove to be effective. In sum, small molecule intervention to modulate enzymatic activities of these proteins has potential therapeutic benefit in NAFLD.

## Concluding remarks

PNPLA3 is an attractive target for treating NAFLD. The variant PNPLA3-I148M increases the risk of NAFLD progression. Mechanistic insights from animal models and mammalian cell lines indicate conspicuous changes in lipid droplet dynamics. Future studies will focus on identifying targets that can modulate PNPLA3 expression or alter its activity to ameliorate NAFLD in patients harboring the disease variant.
